# Relationship between dietary protein intake and serum essential free amino acid concentrations in Japanese pregnant women: an observational study

**DOI:** 10.1186/s12884-025-07962-w

**Published:** 2025-08-14

**Authors:** Takuya Shibasaki, Hirohiko Nakamura, Takuya Kamimura, Fuka Tabata, Satomi Kawakami, Mayumi Inubashiri, Masayoshi Hosaka, Kiwamu Noshiro, Takeshi Umazume, Kazuhiro Miyaji

**Affiliations:** 1 Health Care & Nutritional Science Institute, Morinaga Milk Industry Co., Ltd, Zama, Kanagawa 252-8583 Japan; 2 Department of Public Health, Graduate School of Medicine, Hokkaido University, Sapporo, Hokkaido 060-8638 Japan; 3 Fukuzumi Obstetrics and Gynecology Clinic, Sapporo, Hokkaido 062-0043 Japan; 4 Department of Obstetrics and Gynecology, Hokkaido University Graduate School of Medicine, Sapporo, Hokkaido 060-8648 Japan

**Keywords:** Amino acids, Protein, Pregnancy, Nutrition, Diet, Nutritional status, Mercaptalbumin, Albumin, Tryptophan, Threonine

## Abstract

**Background:**

Insufficient protein intake decreases serum free amino acid (FAA) concentrations and worse whole-body nutritional status, leading to poor pregnancy outcomes. Protein can be obtained from various dietary sources and differs in amino acid composition. However, it remains unclear whether FAA patterns and nutritional status are influenced by differences in dietary protein sources. Therefore, this study aimed to explore the relationship between serum FAAs, type of protein source intake, and protein nutritional status to improve diet during pregnancy.

**Methods:**

In this secondary analysis of data from an observational study of pregnant Japanese women (*n* = 115), we examined the relationship between serum FAA concentrations, the intake of each protein source, and protein nutritional status. Serum FAA concentrations and protein nutritional biomarkers were measured, and the intake of each protein source was estimated using a brief self-administered diet history questionnaire.

**Results:**

Serum FAA concentrations were significantly positively correlated with animal protein intake. Weight-adjusted total and animal protein intakes in the first tertile (T1) of the reduced albumin (Alb) ratio were significantly lower than those in the second and third tertiles (T2-3). Tryptophan concentrations in the second and third trimesters of pregnancy were significantly positively correlated with the reduced Alb ratio. Threonine in T1 of the reduced Alb ratio was significantly lower than that in T2-3 in the second trimester.

**Conclusions:**

Individual FAA concentrations during pregnancy are affected differently according to the dietary protein source and intake. The intake of animal proteins is effective in maintaining essential FAAs during pregnancy. Protein deficiency may lead to decreased concentrations of serum tryptophan, threonine, and protein nutritional biomarkers. Identifying a proper strategy for ensuring adequate protein intake may contribute to healthy birth outcomes.

**Graphical Abstract:**

Inadequate protein intake decreases serum free amino acid concentrations, leading to poor overall nutritional status and poor pregnancy outcomes. Animal protein intake and reduced albumin, a novel indicator of protein nutrition, were significantly associated with serum tryptophan and threonine concentrations. Animal protein intake is effective in maintaining essential free amino acid concentrations during pregnancy and may contribute to healthy birth outcomes. Trp, tryptophan; Thr, threonine; Phe, phenylalanine; Met, methionine; Lys, lysine; BCAAs, branched-chain amino acids; His, histidine.

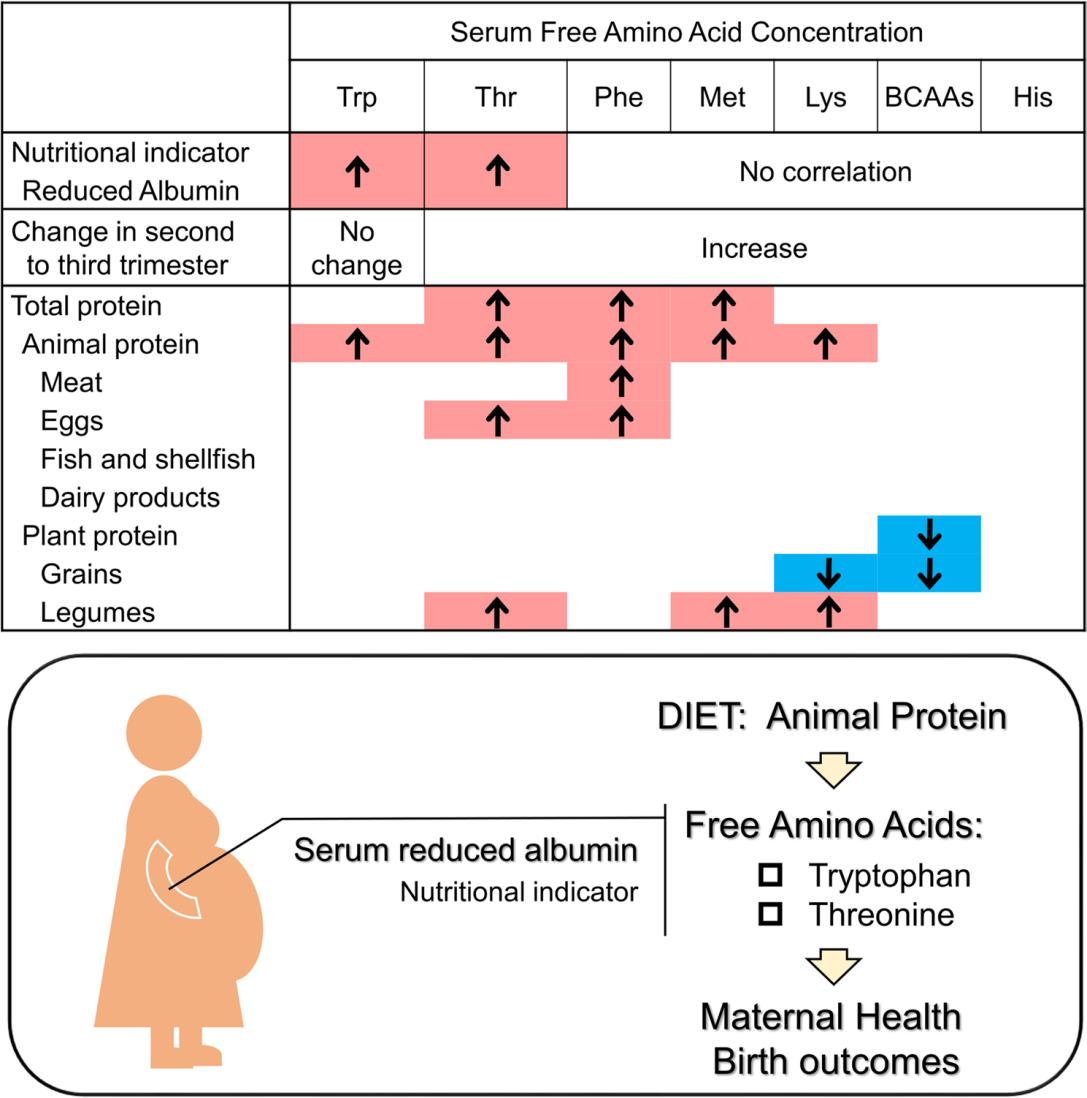

**Supplementary Information:**

The online version contains supplementary material available at 10.1186/s12884-025-07962-w.

## Background

The protein requirement in pregnant women is higher than that in non-pregnant women and increases as gestation proceeds [1, 2]. According to the current dietary reference intakes in Japan, while the recommended daily allowance (RDA) for protein is 50 g/d for non-pregnant women aged 18 years or older, the RDA in the first, second, and third trimesters of gestation are 50 g/d, 55 g/d, and 75 g/d, respectively [3]. Furthermore, recent studies using the indicator amino acid oxidation method have suggested that the estimated average requirement and RDA for proteins may be higher than the respective dietary reference intakes obtained using a traditional nitrogen balance method [1]. Hence, the protein intake of pregnant women may be insufficient, as demonstrated in a Japanese health and nutrition survey [4]. Insufficient protein intake may increase the risk of maternal depression [5], low birth weight [2, 6], and premature birth [7].

Dietary questionnaires and various nutritional biomarkers can be used to assess protein intake. Estimates obtained from self-report questionnaires may result in underestimations or overestimations and should be evaluated together with objective biomarkers. The serum reduced albumin (Alb) ratio to total albumin is a more sensitive biomarker than albumin and transthyretin (TTR) concentrations [8]. A reduced Alb ratio reflects the protein intake in pregnant Japanese women and is associated with infant birth weight [9, 10]. Strategies for protein intake during pregnancy can be proposed to elucidate the relationship between serum free amino acid (FAA) concentrations, dietary protein sources, and protein nutritional status using these biomarkers.

Recent studies have focused on the amino acid composition of each dietary protein source. FAAs decrease in the first trimester and remain at low concentrations throughout gestation [11] as they are concentrated in the placenta for fetal protein synthesis [12]; moreover, they are diluted because of increased blood volume [13]. The concentrations of several amino acids, including valine (Val), leucine (Leu), isoleucine (Ile), phenylalanine (Phe), lysine (Lys), and tryptophan (Trp), are lower in pregnant women than in non-pregnant women [14, 15]. The concentrations of Val, Leu, Ile, and Trp decrease throughout the gestation period [16, 17]. Some risks associated with pregnancy are partly explained by lower concentrations of FAAs, such as Trp [18–20], threonine (Thr) [21], and histidine (His) [20, 22]. FAAs play essential roles in various biological functions, such as protein synthesis, fuel for energy, and precursors for various neurotransmitters [11]. The requirements of several amino acids are increased during pregnancy. The requirements of Lys, glycine, Phe, and tyrosine have been shown to increase in clinical trials [23–26], and the requirements of Thr, Lys, Ile, and Trp have also been found to increase in animal studies [27–30]. Hence, the status of serum FAAs during pregnancy is unique; however, to the best of our knowledge, the relationship between dietary protein intake and serum FAAs has not been fully investigated. Moreover, whether FAA patterns and nutritional status are influenced by differences in dietary protein sources remains to be elucidated.

We hypothesized that maternal FAAs would be positively correlated with protein intake and biomarkers and that these impacts would differ according to the amount and source of dietary protein. This study aimed to elucidate the relationship between serum FAAs, type of protein source intake, and protein nutritional status to improve diet during pregnancy.

## Methods

### Study design and participants

In this secondary analysis of data from an observational study of pregnant Japanese women (*n* = 115), we examined the relationship between serum FAA concentrations, the intake of each protein source, and protein nutritional status. The study design and anthropometric and demographic characteristics of the participants have been described in detail in previous reports [10, 31]. Briefly, pregnant women scheduled to deliver at Fukuzumi Obstetrics and Gynecology Hospital between January and June 2021 were enrolled. The inclusion and exclusion criteria for this study were also based on those of the original study: participants were selected if they were 20 years of age or older at the time of recruitment and had a singleton pregnancy. Pregnant women with multiple gestations, depression, oxidative stress-related diseases (such as hepatic or renal impairment), transfer to other hospitals, or inability to provide a blood sample were excluded. Of the 126 pregnant women who initially agreed to participate in the study, ten were transferred to other hospitals later on, and one was unable to complete the data collection process. As a result, 115 pregnant women were included in the analysis.

### Dietary protein intake

As described in a previous report [10], the dietary intake in the second trimester was estimated using a brief self-administered diet history questionnaire [32]. Protein intake was evaluated separately for each source (grains, meat, fish and shellfish, eggs, dairy products, and legumes). It was categorized as animal- and plant-derived, with animal protein intake comprising meat, fish and shellfish, eggs, and dairy products, and plant-derived protein intake comprising grains and legumes. Grains included rice, bread, and pasta, while legumes included tofu and natto (fermented soybeans), which are mainly composed of soy. Energy adjustments were performed to determine the intake of protein and protein sources using the residual method [33].

### Serum biological parameters

As described in a previous report [10], serum FAAs in the second and third trimester were measured using an automated amino acid analyzer (L-8900, Hitachi High-technologies, Tokyo, Japan). Briefly, serum was mixed with 6.66% trichloroacetic acid to remove proteins. Supernatants were filtered through 0.20-µm polyvinylidene fluoride filters (Thomson Instrument, Oceanside, CA), and the filtrates were applied to the analyzer. The serum reduced Alb ratio (UltiMate 3000 UHPLC, Thermo Fisher Scientific, Tokyo, Japan), Alb (Spotchem EZ SP-4430, Arkray, Kyoto, Japan), and TTR (ELISA kit, Abnova, Taipei, Taiwan) were measured as protein nutritional biomarkers.

### Statistical analysis

Values are expressed as means ± standard deviations. Welch’s *t*-test was performed to compare differences in biochemical parameters and protein intake between the first tertile (T1) and second and third tertiles (T2-3) and between the second and third trimesters. Simple linear regression analysis was performed to examine the relationship between FAAs and the reduced Alb ratio. Multivariate linear regression analyses were performed to examine the relationship between each type of protein intake as an explanatory variable, and each serum FAA as an objective variable. Age and gestation period were used as adjustment variables. All statistical analyses were performed using JMP software version 13.2.1 (SAS Institute, Cary, NC, USA), and statistical significance was set at a *P*-value of less than 0.05.

### Ethics approval and consent to participate

This study was conducted in accordance with the principles of the Declaration of Helsinki and approved by the Institutional Review Board of Hokkaido University Hospital (approval number: 019–0390) and Japan Conference of Clinical Research (approval number: MNS-01). All participants provided written informed consent.

## Results

### Participant characteristics and protein intake

As described in a previous report [10], the mean age of the subjects was 30.6 ± 3.9 years old and their mean weight was 53.1 ± 6.6 kg. Protein intake from animal sources accounted for 54.8 ± 8.0% of the total protein intake (Table 1). Animal protein was significantly higher than plant protein (54.8 ± 8.0% and 45.2 ± 8.0%, respectively, *P* < 0.001). Focusing on the six major protein sources that accounted for 84.1% of total protein intake, the protein intake from each source was 12.4 ± 5.8 g/d for meat, 8.9 ± 4.7 g/d for fish and shellfish, 4.3 ± 2.9 g/d for eggs, 3.8 ± 2.5 g/d for dairy products, 10.8 ± 2.6 g/d for grains, and 4.3 ± 3.0 g/d for legumes.

**Table 1:** Protein intakes of each source and serum amino acids

	Second trimester	Third trimester	*P*-value^*1^
Protein intakes (g/kg/d)^*2^			
Animal protein			
Meat	0.21 ± 0.10		
Fish and shellfish	0.15 ± 0.08		
Eggs	0.07 ± 0.05		
Dairy products	0.07 ± 0.05		
Plant protein			
Grains	0.19 ± 0.05		
Legumes	0.07 ± 0.05		
Amino acids (µM)			
Total FAAs	2103 ± 249	2315 ± 309	**< 0.0001**
Essential FAAs	772 ± 122	862 ± 152	**< 0.0001**
Threonine	171 ± 37	195 ± 46	**< 0.0001**
Valine	158 ± 29	169 ± 34	**0.008**
Methionine	20.8 ± 5.7	24.4 ± 6.2	**< 0.0001**
Isoleucine	40.9 ± 11.7	50.5 ± 12.9	**< 0.0001**
Leucine	74.3 ± 17.9	90.0 ± 21.7	**< 0.0001**
Phenylalanine	60.2 ± 12.1	64.6 ± 12	**0.003**
Tryptophan	36.5 ± 4.8	36.0 ± 6.4	0.77
Lysine	144 ± 27	158 ± 35	**0.001**
Histidine	66.1 ± 9.2	74.3 ± 11.7	**< 0.0001**

Data are presented as means ± standard deviations

*FAAs* free amino acids

^*1^ Significance was demonstrated at a *P*-value of < 0.05 (shown in bold)

^*2^ Energy adjustment was performed for each protein intake using the residual method

### Serum biological parameters

Except for Trp, all essential FAA concentrations were significantly higher in the third trimester than in the second trimester (Table 1). In the second and third trimester, reduced Alb ratios were 82.5 ± 2.2% and 81.4 ± 2.4%, respectively; Alb concentrations were 31.8 ± 2.6 g/L and 30.8 ± 2.3 g/L, respectively; and TTR concentrations were 120 ± 44 mg/L and 158 ± 71 mg/L, respectively.

### Age and body weight in relation to protein Intake and serum FAAs

In simple liner analyses, there were no significant correlation between weight or age and protein intake or essential FAAs (Table 2).

**Table 2:** Simple linear regression analyses of age and body weight with protein intakes and serum amino acids

	Age		Body Weight	
	*R*	*P*-value^*1^	*R*	*P*-value^*1^
Protein intake (g/d)				
Total protein	0.026	0.783	0.086	0.362
Animal protein	0.018	0.853	0.063	0.501
Plant protein	0.021	0.828	0.054	0.570
FAAs (µM)				
Second trimester				
Total FAAs	−0.045	0.631	0.130	0.166
Essential FAAs	−0.149	0.112	0.177	0.059
Third trimester				
Total FAAs	0.069	0.467	−0.059	0.533
Essential FAAs	−0.005	0.959	0.027	0.772

*FAAs* free amino acids

^*1^ Significance was demonstrated at a *P*-value of < 0.05

### Protein intake and protein nutritional biomarkers

Protein intake in the second trimester was compared between tertiles of each of the three protein nutritional biomarkers (Table 3). The total and animal protein intakes in the T1 of reduced Alb ratio were significantly lower than those in T2-3. There were no significant differences in protein intake between tertiles of Alb and TTR.

**Table 3:** Relationship between serum nutritional biomarkers and protein intakes

	First tertile	Second and third tertile	*P*-value^*1^
Reduced Alb (%)	80.2 ± 1.2	83.7 ± 1.6	**< 0.0001**
Number (n)	38	77	
Protein intake (g/kg/d) ^*2^			
Total protein	0.86 ± 0.14	0.94 ± 0.17	**0.022**
Animal protein	0.47 ± 0.13	0.53 ± 0.15	**0.024**
Plant protein	0.40 ± 0.05	0.41 ± 0.08	0.497
Alb (g/L)	28.9 ± 1.0	33.1 ± 1.9	**< 0.0001**
Number (n)	35	80	
Protein intake (g/kg/d) ^*2^			
Total protein	0.91 ± 0.14	0.91 ± 0.18	0.966
Animal protein	0.50 ± 0.13	0.51 ± 0.15	0.736
Plant protein	0.41 ± 0.05	0.40 ± 0.08	0.512
TTR (mg/L)	77.4 ± 14.7	141.7 ± 37.0	**< 0.0001**
Number (n)	38	77	
Protein intake (g/kg/d) ^*2^			
Total protein	0.92 ± 0.15	0.91 ± 0.17	0.677
Animal protein	0.50 ± 0.14	0.51 ± 0.15	0.726
Plant protein	0.42 ± 0.07	0.40 ± 0.07	0.094

Data are presented as means ± standard deviations

*Alb* albumin, *TTR* transthyretin

^*1^ Significance was demonstrated at a *P*-value of < 0.05 (shown in bold); Welch’s t-test

^*2^ Adjusted for body weight

### Reduced Alb ratio and serum FAAs

In the linear regression analyses, Trp showed a significant positive correlation with the reduced Alb ratio in both the second and third trimesters (Table 4). Analysis of the tertiles of the reduced Alb ratio revealed that Thr and Trp in T1 were significantly lower than those in T2-3 in the second trimester, and Trp in T1 was significantly lower than that in T2-3 in the third trimester (Table 5).

**Table 4:** Simple linear regression analyses of serum amino acids vs. serum reduced Alb

	Reduced Alb (%)			
	Second trimester		Third trimester	
	*R*	*P*-value^*1^	*R*	*P*-value^*1^
Total FAAs (µM)	0.046	0.625	0.086	0.361
Essential FAAs	0.099	0.293	0.106	0.260
Threonine	0.131	0.163	0.166	0.076
Valine	0.166	0.076	0.155	0.099
Methionine	0.147	0.118	0.062	0.513
Isoleucine	0.068	0.470	0.063	0.501
Leucine	−0.070	0.459	−0.037	0.696
Phenylalanine	0.025	0.794	0.024	0.802
Tryptophan	0.208	**0.026**	0.288	**0.002**
Lysine	0.010	0.918	0.003	0.975
Histidine	0.045	0.634	0.046	0.625

*FAAs *free amino acids, *Alb* albumin

^*1^ Significance was demonstrated at a *P*-value of < 0.05 (shown in bold)

**Table 5:** Relationship of serum reduced albumin and serum amino acids

	First tertile	Second and third tertile	*P*-value^*1^
Second trimester of gestation period			
Number (n)	38	77	
Reduced Alb (%)	80.2 ± 1.2	83.7 ± 1.6	**< 0.0001**
Total FAAs (µM)	2079 ± 239	2114 ± 254	0.472
Essential FAAs	753 ± 106	781 ± 129	0.227
Threonine	162 ± 29	175 ± 40	**0.040**
Valine	154 ± 29	160 ± 29	0.320
Methionine	19.5 ± 4.1	21.4 ± 6.3	0.051
Isoleucine	40.7 ± 11.2	40.9 ± 12.0	0.914
Leucine	76.7 ± 16.1	73.1 ± 18.6	0.282
Phenylalanine	59.7 ± 10.6	60.5 ± 12.8	0.735
Tryptophan	35.3 ± 4.4	37.1 ± 4.9	**0.044**
Lysine	140 ± 20.6	146 ± 29.2	0.170
Histidine	65.8 ± 10.5	66.2 ± 8.6	0.832
Third trimester of gestation period			
Number (n)	38	77	
Reduced Alb (%)	78.7 ± 1.3	82.7 ± 1.6	**< 0.0001**
Total FAAs (µM)	2273 ± 300	2336 ± 313	0.303
Essential FAAs	840 ± 141	872 ± 157	0.273
Threonine	185 ± 43	200 ± 47	0.086
Valine	164 ± 30	171 ± 35	0.239
Methionine	23.9 ± 6.0	24.7 ± 6.3	0.495
Isoleucine	50.5 ± 12.0	50.5 ± 13.4	0.984
Leucine	92.1 ± 19.7	88.9 ± 22.7	0.431
Phenylalanine	64.0 ± 11.3	64.9 ± 12.2	0.696
Tryptophan	33.7 ± 5.7	37.1 ± 6.5	**0.004**
Lysine	154 ± 32	160 ± 37	0.421
Histidine	73.0 ± 13.0	74.9 ± 11.0	0.451

^*1^ Significance was demonstrated at a *P*-Value of < 0.05 (shown in bold); Welch’s t-test

### Protein intake and serum FAAs

Total protein intake was significantly positively correlated with Thr, methionine (Met), and Phe concentrations (Table 6). Animal protein intake showed a significant positive correlation with total FAAs and essential FAAs, including Thr, Met, Phe, Trp, and Lys. Meat protein intake was significantly and positively correlated with Phe concentrations. Egg protein intake showed a significant positive correlation with Thr and Phe concentrations. Plant protein intake was significantly negatively correlated with Val, Leu, and Ile concentrations. Grain protein intake was significantly negatively correlated with essential FAAs, such as Val, Ile, Leu, and Lys. Legume protein intake showed a significant positive correlation with Thr, Met, Phe, and Lys concentrations. There was no significant association with any of the FAAs for protein intake from fish, shellfish, and dairy products.

**Table 6:** Multivariate linear regression analyses of serum amino acids *vs.* protein intakes

	Independent value^*1*2^									
	Protein intake of each source (g/kg/d)^*3^									
	Total		Animal		Plant		Meat		Fish and shellfish	
	*β*	*P*-value	*β*	*P*-value	*β*	*P*-value	*β*	*P*-value	*β*	*P*-value
Dependent value										
FAAs (µM)										
Total FAAs	0.170	0.071	0.218	**0.020**	−0.043	0.650	0.089	0.351	0.158	0.095
Essential FAAs	0.162	0.082	0.227	**0.014**	−0.082	0.383	0.134	0.153	0.090	0.338
Threonine	0.264	**0.004**	0.255	**0.006**	0.101	0.284	0.124	0.188	0.159	0.091
Valine	0.006	0.952	0.116	0.209	−0.222	**0.016**	0.058	0.534	0.037	0.693
Methionine	0.235	**0.012**	0.189	**0.043**	0.166	0.078	0.053	0.573	0.169	0.074
Isoleucine	0.018	0.851	0.116	0.216	−0.193	**0.038**	0.026	0.780	0.098	0.295
Leucine	−0.019	0.842	0.118	0.210	−0.283	**0.002**	0.078	0.408	0.021	0.828
Phenylalanine	0.238	**0.011**	0.266	**0.004**	0.016	0.867	0.259	**0.005**	0.030	0.753
Tryptophan	0.150	0.102	0.183	**0.046**	−0.019	0.835	0.055	0.553	0.069	0.460
Lysine	0.172	0.069	0.207	**0.028**	−0.017	0.855	0.167	0.078	0.015	0.872
Histidine	0.045	0.633	0.079	0.398	−0.056	0.552	0.018	0.849	0.048	0.609

	Independent value^*1*2^							
	Protein intake of each source (g/kg/d)^*3^							
	Eggs		Dairy products		Grains		Legumes	
	*β*	*P*-value	*β*	*P*-value	*β*	*P*-value	*β*	*P*-value
Dependent value								
FAAs (µM)								
Total FAAs	0.140	0.139	0.060	0.528	−0.136	0.156	0.105	0.267
Essential FAAs	0.164	0.079	0.078	0.408	−0.226	**0.016**	0.160	0.087
Threonine	0.194	**0.038**	0.034	0.722	−0.092	0.335	0.215	**0.021**
Valine	0.069	0.456	0.097	0.295	−0.220	**0.018**	−0.010	0.914
Methionine	0.183	0.051	−0.022	0.819	−0.060	0.532	0.243	**0.009**
Isoleucine	0.102	0.278	0.022	0.817	−0.246	**0.009**	0.005	0.957
Leucine	0.122	0.195	0.022	0.815	−0.299	**0.001**	−0.011	0.908
Phenylalanine	0.194	**0.038**	−0.013	0.894	−0.184	0.053	0.211	**0.024**
Tryptophan	0.155	0.091	0.161	0.080	−0.088	0.346	0.121	0.191
Lysine	0.119	0.210	0.119	0.210	−0.267	**0.005**	0.254	**0.007**
Histidine	0.013	0.891	0.115	0.219	0.070	0.464	0.071	0.450

*FAAs* free amino acids

^*1^Significance was demonstrated at a *P*-value of < 0.05 (shown in bold)

^*2^Adjusted for age and gestation period

^*3^Adjusted for body weight

## Discussion

In this study, we examined the relationship between FAAs, intake of each protein source, and protein nutritional status during pregnancy.

Regarding sources of protein intake, animal protein was significantly higher than plant protein, similar to the data from the 2019 National Health and Nutrition Survey in Japan [34]. Serum essential FAA concentrations increased from the second trimester to the third trimester, but only Trp remained unchanged. The mean concentrations of all essential FAAs, except Thr, were lower than those previously reported in healthy volunteers [35, 36]. Compared to the general range of concentrations in healthy Japanese women [37], most essential FAAs were within the reference intervals; however, Trp concentrations were below the lower limit and Thr concentrations were above the upper limit. Hence, Trp concentrations are specifically lower and Thr concentrations are higher in pregnant women compared with non-pregnant women. These observations are consistent with those of previous studies on pregnant American and Belgian women [14, 38]. “The Trp depletion theory” has been proposed, in which immunosuppression by Trp depletion is paramount for preventing allogeneic fetal rejection during pregnancy [39]. In contrast, the “tryptophan utilization theory” has recently been proposed, in which an increase in kynurenine metabolism through tryptophan utilization is crucial for pregnancy maintenance [40, 41]. Furthermore, low Trp concentrations during pregnancy are associated with postpartum depression [18, 42], sleep disorders [43], preterm birth [19], and inflammatory responses [42]. Therefore, it remains inconclusive whether Trp reduction is necessary during pregnancy. Instead, its utilization rather than depletion appears to be of importance. In human studies using the acute Trp depletion method, diets with critically low Trp concentrations reduced brain function and impaired the serotoninergic system [44, 45]. Therefore, insufficient protein intake during pregnancy may decrease Trp concentrations and increase the risk of disease in both the mother and fetus. While the mechanism remains unclear, low Trp concentration could be attributed not only to a physiological response but also to inadequate protein intake during pregnancy. The mechanism underlying the increase in Thr concentrations during pregnancy also remains unclear. In animal studies, Thr requirement increased during late pregnancy [27]. In clinical trials, Thr concentrations during pregnancy were negatively correlated with preterm delivery [21]. Therefore, increased Thr concentrations may contribute to pregnancy maintenance.

We investigated the associations between maternal age and body weight with total, animal-based, and plant-based protein intake, as well as total and essential FAAs. No statistically significant correlations were observed among these variables. Aging is associated with reduced food intake and FAAs [46]. As previously reported [10], the limited age variability in our study (30.6 ± 3.9 years) may account for the absence of associations. Some amino acids are elevated in obese pregnant women and may be associated with maternal weight gain and birth weight [47]. However, this association remains unclear in healthy pregnant women. In this study, the fact that fewer than 1% of participants had a BMI ≥ 30 may have influenced the analytical results. Further investigation is needed to clarify these relationships.

We examined the relationship between protein intake and tertiles of each protein biomarker to determine which biomarkers were associated with protein intake. Serum reduced Alb ratio, Alb concentration, and TTR concentration have been reported previously [10]. Moreover, previous reports have suggested　that only the reduced Alb ratio, but not Alb or TTR concentrations, reflects protein intake during pregnancy, using multivariable linear regression analyses [10]. In the present study, total and animal protein intakes were higher in T2-3 of the reduced Alb ratio than in T1; however, Alb and TTR showed no correlation. The finding that the serum reduced Alb ratio reflects protein nutritional status supports the results of a previous report [10]. Additionally, we examined the relationship between the reduced Alb ratio and serum essential FAAs to identify FAAs associated with protein nutritional status. The results showed that the reduced Alb ratio was positively correlated with only Trp in both the second and third trimesters, suggesting a potential association with insufficient protein intake. If Trp concentrations are sufficient, a linear correlation with reduced Alb concentrations may not be observed because of the ceiling effect. The fact that only Trp did not increase among the essential FAAs indicates that Trp is an amino acid with a particularly high risk of deficiency.　Next, the FAAs were compared between tertiles of the reduced Alb ratio. Comparing T1 with T2-3, Trp and Thr in the second trimester and only Trp in the third trimester were significantly different. There was a significant difference between tertiles only in the second trimester in Thr, suggesting that Thr may be an amino acid of concern for deficiency in the second trimester.

We examined the relationship between protein intake and FAAs to identify the FAAs that are sensitive to dietary protein intake. Trp concentrations positively correlated with animal proteins; Thr concentrations positively correlated with animal, eggs, and legumes proteins. These protein sources are reportedly useful for healthy birth outcomes [7, 48, 49]. In this study, there was no relationship between the outcomes (gestational length and infant birth weight) and FAAs, which may be attributed to the fact that the majority of the population comprised healthy pregnant women. Our study is the first to suggest that, even in a healthy population, various protein sources may have different effects on FAAs. Therefore, insufficient protein intake may worsen pregnancy outcomes in severely undernourished populations. Similarly, for other FAAs, each protein source was affected differently. Lys, Val, Leu, and Ile (branched-chain amino acids) were negatively correlated with plant and grain proteins. As rice and bread are the main staple foods in Japan, pregnant women who consume a high-grain diet may be at risk of decreased FAAs through high-carbohydrate and low-protein diets. Grain intake was positively correlated with carbohydrate intake and negatively correlated with animal protein intake. In addition, it has been suggested that the negative correlation between Lys and grains is due to the amino acid composition of grains, which contain less Lys than animal proteins [50, 51]. The finding that branched-chain amino acids (BCAAs) were negatively correlated with plant proteins was consistent with the findings of previous reports [52]. Lys and BCAAs decrease during protein deficiency and may influence fetal developmental delays in pigs [53]. BCAAs supplementation during maternal food restriction prevents hypertension in adult rat offspring [54]. Further research is needed to confirm their role in human health, but these amino acids may support infant development. His concentrations did not correlate with protein intake. This may be due to the enhanced catabolism of hemoglobin and carnosine to compensate for a deficiency in His in diets for long periods [55]; moreover, His is abundant in common Japanese meals [56]. Therefore, His intake was sufficient for the healthy population in the present study. This may be attributed to the fact that animal protein intake accounts for more than half of the total protein intake in Japanese people and is rich in amino acids [50, 51]. In contrast, plant proteins are consumed in smaller amounts than animal proteins and have lower amino acid scores and digestibility [50]. In summary, serum FAAs are influenced by different sources of dietary protein, and animal protein intake may prevent the deficiency of essential FAAs, such as Trp and Thr, during pregnancy, supporting maternal and fetal health.

This study had a few limitations. First, serum FAAs and protein intake were not estimated before pregnancy or in the first trimester. These cannot be estimated using the brief self-administered diet history questionnaire because the maternal diet is greatly affected by morning sickness and changes in physical condition in T1 [57]. Therefore, we focused on the second and third trimester. Second, unknown factors, including pregnancy complications such as increased blood volume, edema, and hormonal changes, independently affect FAAs. Moreover, hormones, such as insulin, glucagon, and cortisol, also alter FAAs [36]. Third, considering the health risks to participants, blood samples were taken during consultation. Therefore, the time of blood collection could not be standardized, and the effects of diurnal variations could not be eliminated. Fourth, the effects of protein sources not included in the analysis of this study cannot be considered. Our analysis focused on the top 6 major food intakes. Vegetable protein intakes were low compared with other reported protein sources (0.02–0.03 g/kg/d), and protein intakes from certain foods, such as nuts, could not be assessed because they were not included in the brief self-administered diet history questionnaire. As noted above, plant proteins are generally considered to be difficult to digest and many contain low levels of limiting amino acids such as Lys [48]. However, it has been suggested that limiting amino acids can be compensated for by combining different protein sources [48], and the contribution of plant proteins could be higher when the combined effect of these sources is considered. Finally, the optimal FAA concentrations during pregnancy remain unclear. It is necessary to investigate the intake of each amino acids and clarify whether it meets the intake requirements of pregnant women. In future studies, healthy pregnancy outcomes and disease risk should be examined using larger sample sizes to identify useful strategies for adequate protein nutrition.

## Conclusion

Individual FAA concentrations during pregnancy are affected differently by the dietary protein source and intake. The intake of animal proteins is effective in maintaining essential FAAs. Protein deficiency may lead to decreased concentrations of serum Trp, Thr, and protein nutritional biomarkers. Identifying a proper strategy for protein intake may contribute to healthy birth outcomes for mothers and babies.

## Supplementary Information

Supplementary Material 1.

## Data Availability

De-identified individual participant data that support the findings of this study are available upon reasonable request, pending application and approval.

## Abbreviations

Alb: Albumin
BCAAs: Branched-chain amino acids
FAAs: Free amino acids
His: Histidine
Ile: I soleucine
Leu: Leucine
Lys: Lysine
Met: Methionine
Phe: Phenylalanine
Thr: Threonine
Trp: Tryptophan
TTR: Transthyretin
Val: Valine
