# Supplementary material for: Relationship between dietary protein intake and serum essential free amino acid concentrations in Japanese pregnant women: an observational study
Source: BMC Pregnancy Childbirth. 2025 Aug 14;25:852. doi: 10.1186/s12884-025-07962-w (PMC12351771; doi:10.1186/s12884-025-07962-w)
Supplement: Supplementary file 1 — Supplementary Material 1. [file 12884_2025_7962_MOESM1_ESM.docx]

**Supplemental Table 1. Multivariate linear regression analyses of serum amino acids *vs* gestation outcomes.**

|  | Independent value^*1^ | | | |
| --- | --- | --- | --- | --- |
|  | Gestation length (weeks)^*2^ | | Infant birth weight (g)^*3^ | |
|  | *β* | *P*-value | *β* | *P*-value |
| Dependent value |  |  |  |  |
| Second trimester |  |  |  |  |
| Total FAAs (µM) | 0.069 | 0.466 | 0.117 | 0.170 |
| Essential FAAs | 0.083 | 0.389 | 0.102 | 0.247 |
| Threonine | 0.170 | 0.078 | 0.106 | 0.233 |
| Valine | 0.012 | 0.906 | 0.086 | 0.343 |
| Methionine | 0.043 | 0.652 | 0.031 | 0.712 |
| Isoleucine | 0.088 | 0.362 | 0.079 | 0.367 |
| Leucine | 0.065 | 0.499 | 0.113 | 0.196 |
| Phenylalanine | -0.050 | 0.598 | -0.002 | 0.985 |
| Tryptophan | 0.114 | 0.233 | 0.109 | 0.208 |
| Lysine | 0.046 | 0.632 | 0.070 | 0.418 |
| Histidine | -0.005 | 0.953 | 0.057 | 0.496 |
|  |  |  |  |  |
| Third trimester |  |  |  |  |
| Total FAAs (µM) | -0.040 | 0.690 | 0.113 | 0.206 |
| Essential FAAs | 0.061 | 0.536 | 0.085 | 0.339 |
| Threonine | 0.074 | 0.447 | 0.115 | 0.190 |
| Valine | 0.088 | 0.367 | 0.032 | 0.718 |
| Methionine | 0.014 | 0.886 | 0.049 | 0.578 |
| Isoleucine | 0.081 | 0.415 | 0.073 | 0.414 |
| Leucine | 0.037 | 0.709 | 0.095 | 0.287 |
| Phenylalanine | -0.128 | 0.178 | -0.021 | 0.814 |
| Tryptophan | 0.106 | 0.276 | 0.093 | 0.295 |
| Lysine | 0.060 | 0.533 | 0.078 | 0.368 |
| Histidine | -0.040 | 0.671 | -0.016 | 0.852 |

^*1^ Adjusted for maternal age, parity, pre-pregnancy BMI, infant sex, mode of delivery.

^*2^ Adjusted for maternal age, parity, pre-pregnancy BMI, infant sex, mode of delivery, and gestation length.

FAAs, free amino acids; BMI, body mass index.

**Supplemental Table 2. Simple linear regression analyses of major nutrient intakes *vs* protein intakes of each source.**

|  | Three major nutrient intakes (g/kg/d)^*1^ | | | | | | | | | |
| --- | --- | --- | --- | --- | --- | --- | --- | --- | --- | --- |
|  | Protein | | | | | | Fats | | Carbohydrates | |
|  | Total | | Animal | | Plant | |  |  |  |  |
|  | *R* | *P*-value | *R* | *P*-value | *R* | *P*-value | *R* | *P*-value | *R* | *P*-value |
| Protein intakes (g/d/kg) |  |  |  |  |  |  |  |  |  |  |
| Meat | 0.526 | **<.0001** | 0.632 | **<.0001** | -0.054 | 0.570 | 0.497 | **<.0001** | -0.265 | **0.004** |
| Fish and shellfish | 0.450 | **<.0001** | 0.471 | **<.0001** | 0.095 | 0.312 | 0.137 | 0.145 | -0.115 | 0.222 |
| Eggs | 0.524 | **<.0001** | 0.476 | **<.0001** | 0.258 | **0.005** | 0.424 | **<.0001** | -0.135 | 0.150 |
| Milk | 0.299 | **0.001** | 0.389 | **<.0001** | -0.089 | 0.347 | 0.417 | **<.0001** | 0.030 | 0.754 |
| Cereals | -0.142 | 0.130 | -0.328 | **<.0001** | 0.331 | **0.0003** | -0.284 | **0.002** | 0.669 | **<.0001** |
| Legumes | 0.580 | **<.0001** | 0.338 | **<.0001** | 0.665 | **<.0001** | 0.272 | **0.003** | -0.103 | 0.274 |

^*1^ Significance was demonstrated at a *P*-value of < 0.05 (shown in bold).
